# Data on fatty acid profile, optical properties and oxidative stability of sunflower oils used in the treatment of skin wounds

**DOI:** 10.1016/j.dib.2023.109009

**Published:** 2023-02-23

**Authors:** Kátia Flávia Rocha, Elaine Silva de Pádua Melo, Carla Maiara Lopes Cardozo, Rita de Cássia Avellaneda Guimarães, Karine de Cássia Freitas, Mateus Lotério Coelho, Carlos Alberto do Nascimento Ramos, Lincoln Carlos Silva de Oliveira, Leandro F. Cavalheiro, Valter Aragão do Nascimento

**Affiliations:** a Group of Spectroscopy and Bioinformatics Applied Biodiversity and Health (GEBABS), School of Medicine, Federal University of Mato Grosso do Sul, Campo Grande/MS, Brazil, S/N, Campo Grande, 79070-900, Brazil; b Graduate Program in Health and Development in the Central-West Region of Brazil, Federal University of Mato Grosso do Sul, Campo Grande 79079-900, Brazil; c Faculdade de Medicina Veterinária e Zootecnia da Universidade Federal de Mato Grosso do Sul, Campo Grande 79079-900, Brazil; d Chemistry Institute, Federal University of Mato Grosso do Sul, Campo Grande 79070-900, MS, Brazil

**Keywords:** Essential, Sunflower oil, Thermal stability, Antioxidants, Spectrophotometry

## Abstract

This dataset describes the analysis of aflatoxins, macroelement and microelement concentration, oxidative stability and fatty acid profile of infant formula milk powder. Gas chromatography (CG) was used to identity 14 fatty acid methyl esters in in five samples of oils. The Racimat 893 method (induction times), Thermogravimetry (TG), Derivative Thermogravimetry (DTG) and Differential Scanning Calorimetry (DSC) were used to estimate the oxidative stability of oils. In addition, UV-VIS spectroscopic techniques were employed to obtain graphs of the absorption of each oil. The data presented can be useful in identifying compounds available in oils used to promote wound healing and understand the degradation mechanism.

**Specifications Table**

**Table:** 

Subject	Biochemistry

Specific subject area	Chemistry, medicine
Type of data	Table Graph Figure
How the data were acquired	The fatty acid methyl esters (FAMEs) were analyzed by gas chromatography (Thermo Fisher Scientific (FOCUS GC)) to obtain their individual peaks. The oxidative stability of the oils was evaluated by Rancimat 893 method (Metrohm Co, Basel). The 893 Professional Biodiesel Rancimat is an analysis system for easy and safe determination of the oxidation stability of biodiesel (fatty acid methyl ester, FAME) and biodiesel blends in accordance with EN 14112, EN 15751 and EN 16568 standards. Thermogravimetry (TG), Derivative Thermogravimetry (DTG) and Differential Scanning Calorimetry (DSC) curves were performed using TGA Q-50 equipment (TA Instruments, New Castle, DE, USA) and DSC-Q20 equipment, coupled to an RCS90 refrigeration system (TA Instruments, New Castle, DE, USA). Graphs were obtained using Universal Analysis Software. Ultraviolet-Visible and Visible absorption spectra were obtained using a Multiskan SkyHigh Microplate Spectrophotometer (Thermo Scientific). The model operates exclusively through the SkanIt PC software.
Data format	Raw Analyzed
Description of data collection	The fatty acid methyl esters (FAMEs) were prepared with a derivatization solution of ammonium chloride, methanol and sulfuric acid. We used gas chromatography to identify 14 FAMEs present in five samples of essential oils used in wound care. Preparation of the oil samples to analysis by RancimatⓇ: An aliquot of 3.0 ± 0.1g was collected from each oil sample and individually dissolved in deionized water. Thus, the oxidative stability of the five oils was expressed as the oxidative induction period (IP, hrs) measured at 110°C. To obtain the thermogravimetric analysis curves, approximately 8.1 mg of oils were heated from 30 to 600°C under nitrogen flow (60 ml/min), heating rate of 10°C/min. For the differential scanning calorimetry (DSC), the DSC curves were obtained using approximately 8.1 mg of oil under nitrogen atmosphere with a flow rate of 50 mL/min, heating/cooling rate of 20 °C/min in heating cycles and subsequently cooling in temperature ranges from 40° C to – 85 °C. Five graphs of the absorption of the five oils were obtained from the UV-VIS points.
Data source location	Institution: School of Medicine, Federal University of Mato Grosso do Sul, Campo Grande/MS, Brazil. City: Campo Grande, Mato Grosso do Sul, Midwest region. Country: Brazil.
Data accessibility	Repository name: Mendeley data Data identification number: DOI:10.17632/6gthk778xm.2 Direct URL to data: https://data.mendeley.com/datasets/6gthk778xm/2

## Value of the Data

• The data presented in this article indicate that there are differences in the composition of fatty acids, as well as differences in the oxidation time and thermal stability of the medicinal oils used in the treatment of wounds.
• From the data presented in this article, the researchers are able to know the thermal and oxidative stability and especially the composition of fatty acids present.
• The data obtained on of composition of fatty acids, as well as differences in the oxidation time and thermal stability of the oils can be compared with data reported in the Ref. [1]. These data can also be used in comparative studies about medicinal oils use.
• Data on the oxidative stability of essential fatty acid oil can be compared with the international standard EN14112 for samples of sunflower and vegetable oil.

## 1 Objective

In several countries, data on the chemical composition and physical properties of various oils used in the treatment of wounds are scarce. The objective of this experiment was to generate a dataset on fatty acid profile, optical properties and oxidative stability of sunflower oils used in the treatment of skin wounds.

## 2 Data Description

**Fig. 1:**
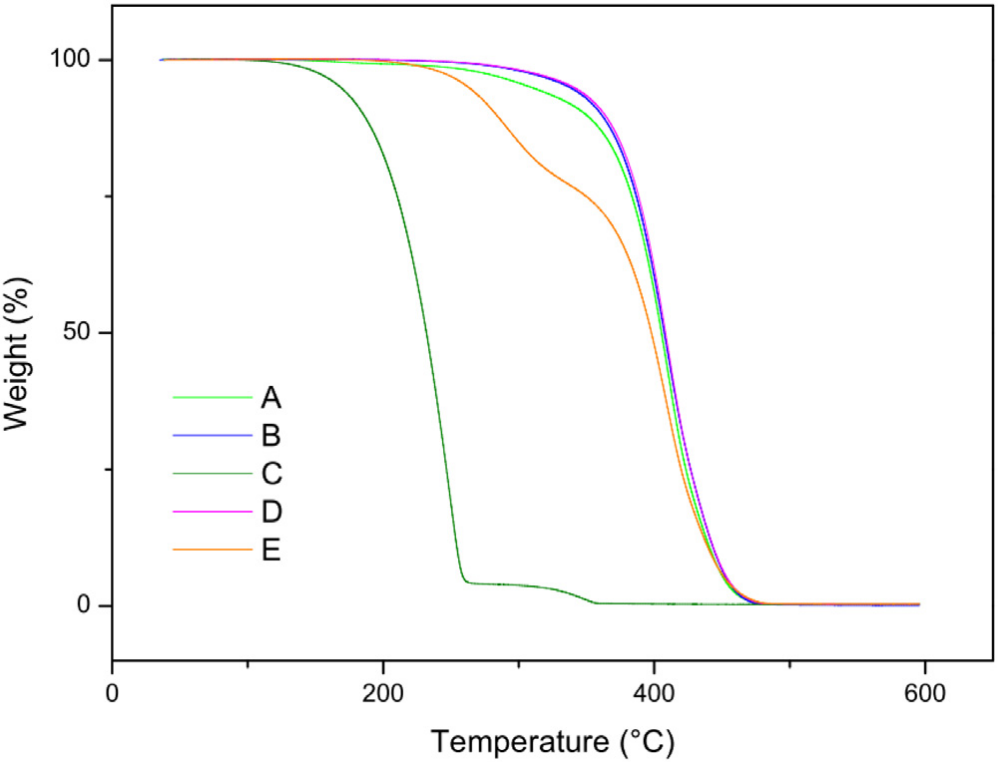
Thermogravimetric analysis (TGA) curves of the: A) Dauf ProtectⓇ oil; B) Derma StarⓇ oil; C) DersolⓇ oil; D) Moph DermeⓇ oil and E) Needs CareⓇ in a nitrogen atmosphere.

**Fig. 2:**
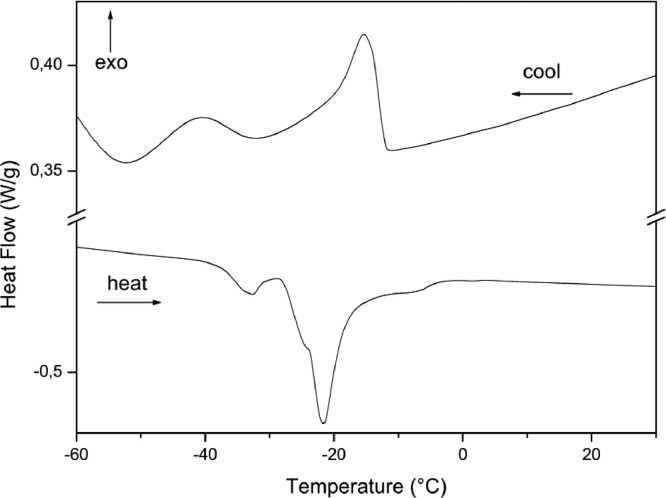
Differential Scanning Calorimetry (DSC) curve of Dauf ProtectⓇ oil in a nitrogen atmosphere.

**Fig. 3:**
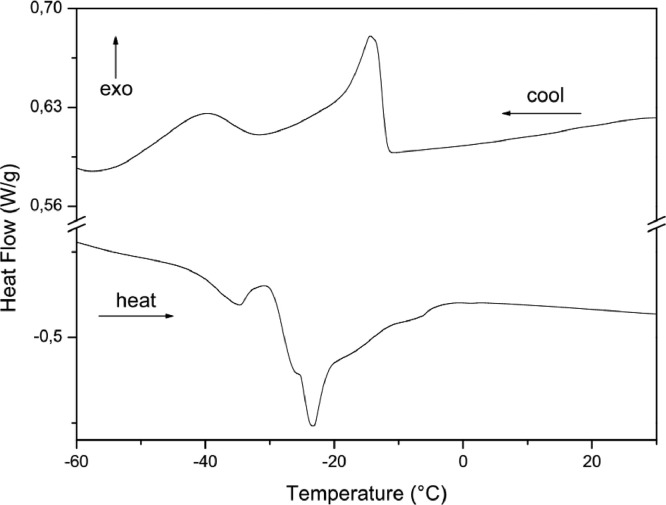
Differential Scanning Calorimetry (DSC) curve of Derma StarⓇ oil in a nitrogen atmosphere.

**Fig. 4:**
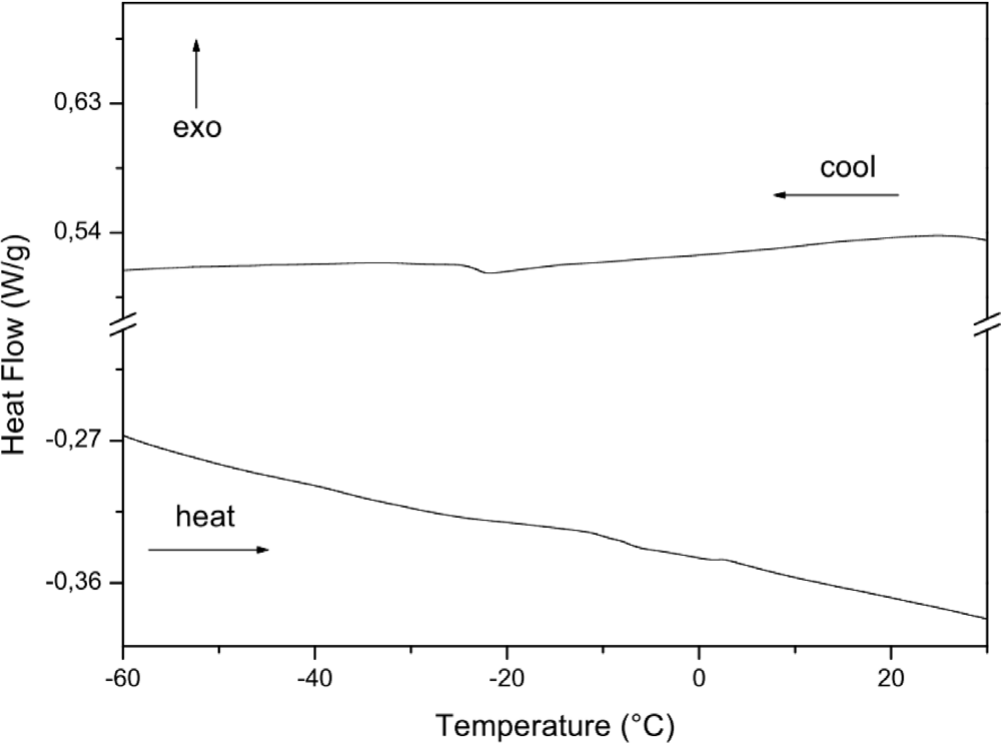
Differential Scanning Calorimetry (DSC) curve of DersolⓇ oil in a nitrogen atmosphere.

**Fig. 5:**
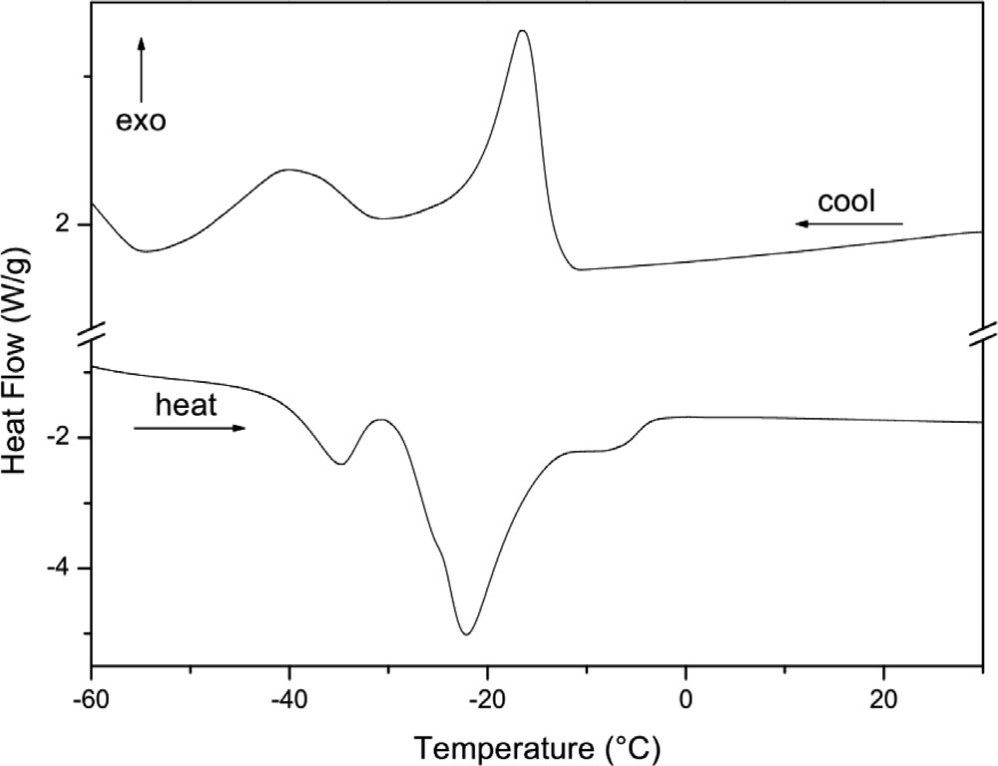
Differential Scanning Calorimetry (DSC) curve of Moph DermeⓇ oil in a nitrogen atmosphere.

**Fig. 6:**
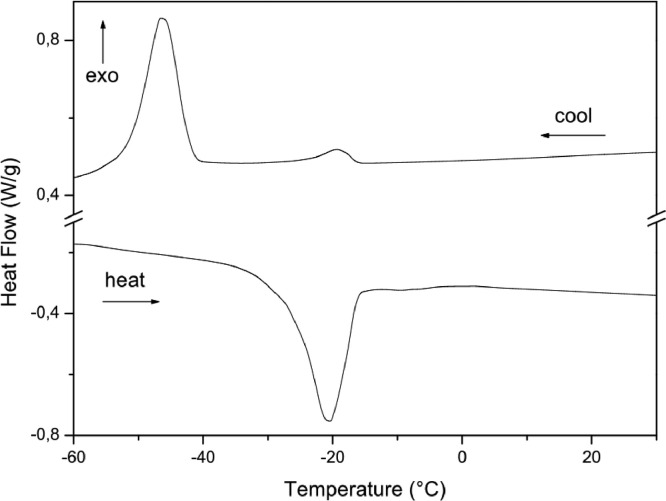
Differential Scanning Calorimetry (DSC) curve of Needs CareⓇ oil in a nitrogen atmosphere.

**Fig. 7:**
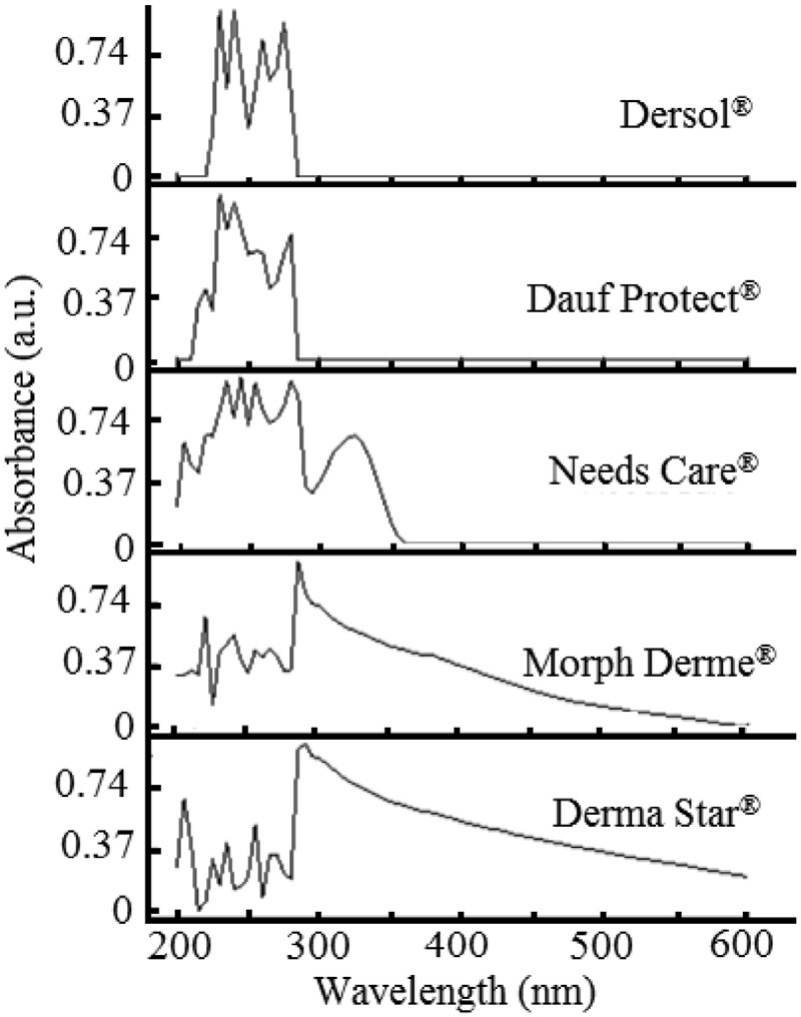
UV-vis absorption spectra of absorbance versus wavelength from 200–600 nm for the DersolⓇ, Dauf ProtectⓇ, Needs CareⓇ, Morph DermeⓇ and Derma StarⓇ oils.

The paper is structured as follows: Section 1.1 (Table 1) shows the experimental data on fatty acids composition of oils Dauf ProtectⓇ, Moph DermeⓇ, Needs CareⓇ, Derma StarⓇ and DersolⓇ, all obtained by CG analysis. In subsection 1.2 (Table 2) we presented data on oxidative stability of the five oils expressed as the oxidative induction period (IP, hours) obtained by Rancimat method. The subsection 1.3 provides data on thermogravimetry (TG), derivative thermogravimetry (DTG) (Fig. 1) and differential scanning calorimetry (DSC) (Table 3, Fig. 2, Fig. 3, Fig. 4, Fig. 5, Fig. 6). The latter data (subsection 1.4, Fig. 7) includes the graph on UV-VIS of absorbance versus wavelength of the Dauf ProtectⓇ, Moph DermeⓇ, Needs CareⓇ, Derma StarⓇ and DersolⓇ oils.

**Table 1:** Fatty acids composition of Dauf ProtectⓇ, Moph DermeⓇ, Needs CareⓇ, Derma StarⓇ and DersolⓇ oils.

Oils					
Fatty acids (%)	Dauf ProtectⓇ	Moph DermeⓇ	Needs CareⓇ	Derma StarⓇ	DersolⓇ
caprylic (C8:0)	4.15	1.99	19.62	1.18	ND
capric (C10:0)	2.40	1.11	9.82	0.63	ND
lauric (C12:0)	0.63	0,08	0.11	ND	ND
myristic (C14:0)	0.07	0.07	0.06	0.08	ND
palmitic (C16:0)	7.96	8.63	ND	ND	12.27
palmitoleic (C16:1)	0.07	0.07	ND	0.08	ND
margaric (C17:0)	0.6	0.07	ND	0.07	ND
stearic (C18:0)	3.22	3.81	ND	ND	ND
oleic (C18:1n9)	25.11	20.96	19.71	ND	44.01
linoleic (C18:2n6c)	48.04	54.87	39.64	50.79	28.01
γ-linolenic (C18:3n6)	0.16	0,30	ND	0.36	ND
α-linolenic (C18:3n3)	3.55	3.43	0.27	4.60	ND
gondoic (C20:1)	0.18	0.19	0.11	0.23	ND
dihomo-γ-linolenic (C20:3n3)	0.51	0.49	0.47	0.46	2.66
Σ SFA	19.66	15.76	29.61	1.96	12.27
Σ MUFA	25.36	21.03	19.71	0.08	44.01
Σ PUFA	52.26	59.09	40.38	56.21	30.67

ND = not determined; Σ SFA = Sum of Saturated fatty acids; Σ MUFA= Sum of Monounsaturated fatty acids (MUFA); Σ PUFA = Sum of Polyunsaturated fatty acids (PUFA)

**Table 2:** Rancimat at 110°C of the Moph DermeⓇ, Needs CareⓇ, Derma StarⓇ, DersolⓇ, Dauf ProtectⓇ oils

	Moph DermeⓇ	Needs CareⓇ	Derma StarⓇ	DersolⓇ	Dauf ProtectⓇ
Rancimat [h]	6.86 ± 0.15	3.93 ± 0.45	6.41 ± 0.53	3.92 ± 0.13	16.38 ± 3.58

Mean ± SD: Standard deviation values are expressed as mean of samples analyzes in duplicates.

**Table 3:** Temperature range, mass losses and residues of the thermal decomposition curves

Oils	Temperature (°C)		Mass Loss (%)	Residue (%)
	Initial	final		
Dauf ProtectⓇ	368.0	480.3	99.56	0.44
Derma StarⓇ	370.8	480.7	99,71	0.29
DersolⓇ	195.3	361.6	99.50	0.49
Moph DermeⓇ	375.7	485.6	99.65	0,35
Needs CareⓇ	248.3	485.6	99.60	0.40

### 2.1 Data obtained by analysis by CG

Data on chromatographic analysis used to estimate the percentages of saturated fatty acids (SAF), monounsaturated fatty acids (MUFA) and polyunsaturated fatty acids (PUFA) of the oils examined in this article are in Table 1. In addition, the fatty acids obtained were the following: caprylic (CH_3_(CH_2_)_6_COOH, C8:0), capric (CH_3_(CH_2_)_8_COOH, C10:0), lauric (CH_3_(CH_2_)_10_COOH, C12:0), myristic (CH3(CH_2_)_12_COOH, C14:0), palmític (CH_3_(CH_2_)_14_COOH, C16:0), palmitoleic (CH_3_(CH_2_)_5_CH=CH(CH_2_)_7_COOH, C16:1), margaric (CH_3_(CH_2_)_15_COOH, C17:0), stearic (CH_3_(CH_2_)_16_COOH, C18:0), oleic (CH_3_(CH_2_)_7_CH=CH(CH_2_)_7_COOH, C18:1n9), linoleic (CH_3_(CH_2_)_4_CH=CHCH_2_CH=CH(CH_2_)_7_COOH, C18:2n6c), γ-linolenic (CH_3_(CH_2_)_4_CH=CHCH_2_CH=CHCH_2_CH=CH(CH_2_)_4_COOH, C18:3n6), α-linolenic (CH_3_CH_2_CH=CHCH_2_CH=CHCH_2_CH=CH(CH_2_)_7_COOH, C18:3n3), gondoic (CH3(CH2)7CH=CH(CH2)9COOH,C20:1), dihomo-γ-linolenic (CH3(CH2)4CH=CHCH2CH=CHCH2CH=CH(CH2)6COOH, C20:3n3).

### 2.2 Data on oxidative stability assessment by the Rancimat method

The oxidative stability index of oils (induction period time) was given in Table 2. The Rancimat induction time at 110°C ranged from 3.92 ± 0.13 to 16.38 ± 3.58 h for the oils studied (Table 2).

### 2.3 Thermogravimetry (TG), derivative thermogravimetry (DTG) and differential scanning calorimetry (DSC)

The data on Thermogravimetry (TG) of the oils are shown in Table 3 and Fig. 1. The data in Table 3 show the thermal decomposition steps of Dauf ProtectⓇ, Derma StarⓇ, DersolⓇ, Moph DermeⓇ and Needs CareⓇ oil.

In addition, the Differential Scanning Calorimetry (DSC) data for the five oils are shown in Table 4 and Fig. 2, Fig. 3, Fig. 4, Fig. 5, Fig. 6. The peaks, temperatures and enthalpies (energies, ΔH) of the various oils tested are in Table 4, while Fig. 2, Fig. 3, Fig. 4, Fig. 5, Fig. 6 show the peaks of the DSC (cooling and heating) curve of undegraded and degraded oils at all levels of mass loss.

**Table 4:** Data on differential Scanning calorimetry of Dauf ProtecⓇ, Derma StarⓇ, DersolⓇ, Moph DermeⓇ and Needs CareⓇ oils.

Samples	Curve	Peak 1			Peak 2		
		Initial temperature (°C)	Peak maximum temperature (°C)	Energy (J/g)	Initial temperature (°C)	Peak maximum temperature (°C)	Energy (J/g)
Dauf ProtectⓇ	Cooling	-12.14	-15.32	4.50	-34.08	-41.98	1.63
	Heating	-37.82	-32.82	4.30	-25.00	-21.76	30.08
Derma StarⓇ	Cooling	-11.84	-14.32	6.17	-33.50	-41.85	3.32
	Heating	-42.06	-35.39	5.65	-27.32	-23.36	45.44
DersolⓇ	Cooling	—–	—–	—–	—–	—–	—–
	Heating	—–	—–	—–	—–	—–	—–
Moph DermeⓇ	Cooling	-13.25	-16.25	50.83	-32.07	-41.00	28.87
	Heating	-41.14	-35.22	63.41	-27.33	-22.30	285.86
Needs CareⓇ	Cooling	-16.36	-19.37	2.22	-42.01	-46.62	29.84
	Heating	-32.19	-20.88	42.31	—–	—–	—–

### 2.4 Data on molecular absorption (UV-VIS)

Fig. 7 shows typical plots of UV-Vis absorption spectra of the five oils from 200 to 600 nm. In addition, the oil samples used for wound treatment showed absorption from 232 to 270 nm. Concerning the absorbance region at 232 nm and 270 nm, it is possible to know the quality of oils, oxidation and adulteration of vegetable oils [2,3]. As it is shown in Fig. 7, the UV region (315-330 nm) is characterized by high signal to noise ratio and high sensitivity, due to the presence of signals from tocopherols and phenolic compounds (270–330 nm) [4]. Low-intensity emission bands at 350-600 nm provides information about polyphenols and other important molecules [5].

## 3 Experimental Design, Materials and Methods

### 3.1 Material

Oils of five manufacturers were purchased from local pharmacies in the city of Campo Grande, Brazil. Total two samples of one brand of each sample were taken for study. In this way total 15 samples (3 samples for Dersol [Manufacturer: BellaPhytus Ltda], 3 samples for Dauf ProtectⓇ [Manufacturer: Dauf Ltda], 3 samples for Needs CareⓇ [Manufacturer: Needs Care Ltda], 3 samples for Morph DermeⓇ [Manufacturer: France Pharma Ltda] and 3 Derma StarⓇ oil [Manufacturer: Pharme Star Comésticos], respectively) were collected for study considering the same batch reference number and date of manufacture.

### 3.2 Gas chromatographic (CG) method

In the present study, for sample preparations the following procedure was performed: a) an amount of 0.16 g of the oils (DersolⓇ, Dauf ProtecⓇ, Needs CareⓇ, Morph DermeⓇ and Derma StarⓇ) were weighed separately, then, the esterification process occurred with the combination of 4 ml of KOH (5%) with the addition of MeOH to each sample; b) Oil samples were placed in a hot water bath and heated to 95°C; c) after cooling, 5 mL of NH_4_Cl-H_2_SO_4_-MeOH (0.5:10:89.5 w:v:v) was added in each oil sample; d) after cooling, each sample was individually homogenized after the addition of 4 ml of saturated NaCl solution; e) subsequently, 5 ml of hexane was added to the oil samples and then homogenized again using a vortex mixer; f) volume of 1 µL of each oil sample was used by the chromatography equipment.

Gas chromatography (GC) analysis was carried out in Thermo Fisher Scientific (FOCUS GC) GC equipment, equipped with split/splitless injector inlet and a flame ionization detector (FID), capillary column DB-Wax (30 m length, 0.32 mm internal diameter) and 0.25 μm of film (J & W Scientific). The temperatures of the injector, column temperature and temperature of the detector were programmed at 250°C, 180°C and 260°C, respectively. The ramp rate was operated of 2°C/min up to 220°C. Hydrogen at a flow rate of 1.0 mL/min was employed as the carrier gas. Nitrogen makeup gas at 20 mL/min was used to minimize band broadening.

For identification, the retention times of the fatty acids were compared to those of standard methyl esters (Sigma-Aldrich, St. Louis, MO, USA). The identification was performed by area normalization, expressing the result in percentage of area of each acid over the total area of fatty acids (%).

### 3.3 Accelerated oxidation tests: the Rancimat method

The accelerated oxidation tests of the oils was evaluated by mean of the RancimatⓇ method (RancimatⓇ 893, Metrohm Co, Basel) [6]. Samples of 3.0 g of oil with Volatile oxidation products were stripped from the oil and dissolved in deionized water. With the Rancimat method (EN 14112, European Norm), the oil samples were exposed to an air flow of 10 L/h and a constant temperature of 110°C. The method allows a continuous recording, that is, draws a curve whose inflection point marks the period of induction, linked to the increase of volatile oxidation products of oils. Thus, stability was expressed as the oxidative induction period (IP, hrs) (see Table 2). All experiments were performed in duplicates for each condition [1].

### 3.4 Thermal analysis: thermogravimetry (TG), derivative thermogravimetry (DTG) and differential scanning calorimetry (DSC)

For thermogravimetric analysis (TG/DTG) of the oils, the TGA Q-50 equipment (TA Instruments, New Castle, DE, USA) was used. The TA Advantage software provided thermal stability curves for the oils. Thus, two types of TG/DTG curves were obtained. Approximately 8.1 mg of oils were added to a platinum crucible under nitrogen atmosphere at a flow rate of 60 mL/min, with temperatures range between 30 °C to 600 °C, and heating rate of 10 °C min^−1^ (Fig. 1, Fig. 2, Fig. 3, Fig. 4, Fig. 5).

DSC analyses were performed at DSC-Q20 equipment, coupled to an RCS90 refrigeration system (TA Instruments). The TA Advantage software provided the DSC curves for the oils. The DSC curves were obtained using approximately 8.1 mg of oil in an aluminum crucibles and with reference a similar crucible empty under nitrogen atmosphere with a flow rate of 50 mL/min, heating/cooling rate of 20 °C/min in heating cycles and subsequently cooling in temperature ranges from 40 °C to -85 °C.

### 3.5 Process of data analysis by UV-VIS

Samples of the DersolⓇ, Dauf ProtectⓇ, Needs CareⓇ, Morph DermeⓇ and Derma StarⓇ oils were diluted separately in HPLC grade hexane at a concentration of 10 g/L. UV-visible absorption measurements were performed using a Multiskan Sky Microplate Spectrophotometer (Thermo Scientific, USA). In this experiment, measurements of photometric signals (absorption spectra) ranged from 200 to 600 nm. A blank analysis was also carried out using the above method. Oil and blank samples were analyzed in triplicate.

## Ethics Statements

The research does not involve using humans and animals as subjects, and the data were not collected from social media platforms.

## CRediT authorship contribution statement

**Kátia Flávia Rocha:** Conceptualization, Investigation, Writing – original draft. **Elaine Silva de Pádua Melo:** Data curation, Visualization, Investigation. **Carla Maiara Lopes Cardozo:** Data curation, Visualization, Investigation. **Rita de Cássia Avellaneda Guimarães:** Data curation, Visualization, Investigation. **Karine de Cássia Freitas:** Data curation, Visualization, Investigation. **Mateus Lotério Coelho:** Data curation, Visualization, Investigation. **Carlos Alberto do Nascimento Ramos:** Data curation, Visualization, Investigation. **Lincoln Carlos Silva de Oliveira:** Data curation, Visualization, Investigation. **Leandro F. Cavalheiro:** Data curation, Visualization, Investigation. **Valter Aragão do Nascimento:** Funding acquisition, Supervision, Writing – review & editing.

## Declaration of Competing Interest

The authors declare that they have no known competing financial interests or personal relationships that could have appeared to influence the work reported in this paper.

## Data Availability

Data on oxidative stability and elemental analysis of Brazilian sunflower oils used as healing agents (Original data) (Mendeley Data).
